# Fluoride in the drinking water and dental caries experience by tooth surface susceptibility among adults

**DOI:** 10.1186/s12903-021-01598-w

**Published:** 2021-05-04

**Authors:** Lina Stangvaltaite-Mouhat, Alina Puriene, Indre Stankeviciene, Jolanta Aleksejuniene

**Affiliations:** 1 Oral Health Centre of Expertise in Eastern Norway, Sørkedalsveien 10A, 0369 Oslo, Norway; 2 Department of Clinical Dentistry, Faculty of Health Sciences, UiT The Arctic University of Norway, 9037 Tromsø, Norway; 3 Institute of Dentistry, Faculty of Medicine, Vilnius University, M. K. Čiurlionis str. 21, 03101 Vilnius, Lithuania; 4 Department of Oral Health Sciences, Faculty of Dentistry, The University of British Columbia, 2199 Wesbrook Mall, Vancouver, Canada

**Keywords:** Adult, Dental caries, Drinking water, Fluorides, Tooth surface susceptibility

## Abstract

**Background:**

Dental caries is the most prevalent non-communicable health condition globally. The surface-based susceptibility hierarchy indicates that surfaces in the same group have similar susceptibility to caries, where the most susceptible group consists of occlusal surfaces of first molars and buccal surfaces of lower first molars, and the least susceptible surfaces are smooth and proximal surfaces of first premolars, canines and incisors. Therefore, fluoride in the drinking water could impact one group more than the other group. The present study examined the association between fluoride levels in the drinking water and dental caries experience in adults in the context of varying tooth surface susceptibility.

**Methods:**

Data from the cross-sectional National Lithuanian Oral Health Survey conducted in 2017–2019 included a stratified random sample of 1398 35–74-year-olds (52% response rate). Dental caries experience in dentine was measured at a surface level. The surfaces were grouped according to their caries susceptibility (group 1 being the most and group 4 the least susceptible), and dental caries experience was calculated separately for each susceptibility group, creating four outcomes. Information about explanatory variable, fluoride levels in the drinking water, was provided by the water suppliers. The questionnaire inquired about potential determinants: sociodemographic characteristics and oral health-related behaviors. Chi-square, Mann–Whitney U and Kruskal Wallis tests were used for descriptive statistics, and linear regression analyses to examine the association between fluoride levels and four outcomes.

**Results:**

The proportions of median decayed, missing, filled surfaces decreased following the surface-based susceptibility hierarchy (group 1–33%, group 2–28%, group 3–24%, group 4–15%). When adjusted for potential determinants, higher-level fluoride (≥ 0.7 ppm vs < 0.7 ppm) in the drinking water associated with lower dental caries experience in all surface-based susceptibility hierarchy groups; Group 1: β = − 0.23 (95 %CI − 0.44; − 0.001), Group 2: β = − 0.44 (95 %CI − 0.82; − 0.07), Group 3: β = − 1.14 (95 %CI − 1.88; − 0.41) and Group 4: β = − 6.28 (95 %CI − 9.29; − 3.30).

**Conclusions:**

The higher-level fluoride in the drinking water associated with lower dental caries experience in adults and this was observed in all surface-based susceptibility groups. However, there is a need to validate the surface-based susceptibility hierarchy in longitudinal adult studies.

## Background

Dental caries has multifactorial etiology; untreated dental caries in permanent teeth is the most prevalent non-communicable health condition and untreated dental caries in deciduous teeth is the 10th most prevalent chronic disease [1, 2]. The reduction in dental caries in industrialized countries during the past several decades was mainly attributed to the introduction of fluorides [3–5]. Smooth and proximal surfaces seem to be less susceptible to caries compared to occlusal surfaces [6]. It was suggested that fluoride mainly prevented smooth surfaces, but it was less effective for preventing caries on occlusal surfaces [7–9].

In addition to categorizing occlusal surfaces as being more and smooth/proximal being less susceptible to caries, a surface-based susceptibility hierarchy to caries has also been suggested, where the most susceptible group consisted of 6 surfaces: occlusal surfaces of first molars and buccal surfaces of lower first molars, the second susceptibility group consisted of 12 surfaces, namely occlusal surfaces of second molars, buccal surfaces of lower second molars, occlusal surfaces of all second premolars and palatal surfaces of upper first molars, the third susceptibility group consisted of 20 surfaces, including occlusal surfaces of first premolars, palatal surfaces of upper lateral incisors, proximal surfaces of first molars, lingual surfaces of lower first molars, buccal surfaces of upper first molars and palatal surfaces of upper second molars, while the least susceptible groups included all other surfaces [10]. This surface-based hierarchy of susceptibility to caries was previously examined among 5–16-year-old children [10]. Researchers suggested that if a surface-based hierarchy of susceptibility exists this may contribute towards designing effective preventive strategies because if surfaces in one group behave the same, i.e., have a similar susceptibility, then the resistance could be improved by applying targeted fluoride treatments.

Not much research attention has been given to this aspect of caries susceptibility. Besides, it is not known if the surface-based susceptibility hierarchy also applies to caries experience among adults. Such knowledge is important because the aging population retains teeth longer and consequently would benefit from cost-effective prevention [11].

We examined the association between fluoride levels in the drinking water and dental caries experience in adults in the context of varying tooth surface susceptibility. The current study tested the following hypotheses: (1) surface-based susceptibility hierarchy groups are related to differences in dental caries experience in adults; (2) fluoride levels in the drinking water and dental caries experience are associated with different surface-based susceptibility hierarchy groups.

## Methods

### Study design and population

The current cross-sectional study analyzed the data from the National Lithuanian Oral Health Survey (NLOHS) conducted in 2017–2019. The NLOHS included a stratified random sample of 35–74 years old subjects from the five largest Lithuanian cities and 10 randomly selected peri-urban/rural areas, one of each of the 10 Lithuanian counties [12]. Those in active military service, in prison, institutionalized, mentally disabled or not present in Lithuania during NLOHS were excluded. Sample size calculation was based on the previous study reporting a 50% recruitment rate [13], which was multiplied by a design effect of 1.5 [14]. In total, 2716 adults were invited, of which 1405 agreed to participate (response rate 52%). Data of 946 (35%) participants were analyzed after excluding those who had missing values.

### Variables and measurements

#### Questionnaire

All participants completed the WHO Oral Health Questionnaire for Adults [15], which was translated from English to an official Lithuanian language and two minority languages: Russian and Polish (and then back to English). The questionnaire was piloted on 10 adults who were not included in the main study.

#### Sociodemographic characteristics

Sociodemographic characteristics included age, sex, education (secondary school or less, higher: college/university less than 4 years, and higher: university 4 years or more), residency (urban, peri-urban/rural), and fluoride levels in the drinking water. Information about the fluoride levels in the drinking water in 15 geographic locations was acquired from the water suppliers. Subsequently, this was dichotomized into the lower-level fluoride < 0.7 ppm and higher-level fluoride ≥ 0.7 ppm in the drinking water [16].

#### Oral health‐related behaviors

Oral health-related behaviors included questions about the frequency of tooth brushing (twice a day or more or once a day or less), use of fluoridated toothpaste (yes, no, don’t know), frequency of consumption of sugar-containing foods, and the time of the last dental visit (12 months ago or less, more than 12 months ago). Consumption of sugar-containing foods was calculated based on responses to eight questions, each of which presented different sugar-containing items, each item reported on a 6-responses scale; ‘1’ rarely/never, ‘2’ several times a month, ‘3’ once a week, ‘4’ several times a week, ‘5’ every day, ‘6’ several times a day. Subsequently, responses were summed up into a total score that ranged from 8 to 40. Based on quartiles of the total score, participants were categorized as having low (scores 8–15), moderate (scores 16–23), and high sugar consumption (scores 24–40).

#### Dental caries experience

The WHO criteria were used to register the D_3_MFS score based on 28 teeth, i.e. without the inclusion of third molars [15]. The dental examination was performed as recommended by the WHO Oral Health Survey Basic Methods 5th Edition [15]; it took place in dental offices using a dental chair, a dental unit light, a probe, and an oral mirror. One trained and calibrated examiner (IS) recorded dental caries experience at a surface level (decayed, missing, filled surfaces [D_3_MFS] score). The intra-examiner agreement was assessed at a tooth level based on duplicate recordings of 10 randomly selected individuals (280 teeth). The Cohen’s Kappa of 0.957 indicated excellent intra-examiner reliability.

For statistical analyses, all surfaces were grouped into four groups according to the surface-based susceptibility hierarchy as suggested by Batchelor and Sheiham [10]. Group 1 consisted of six types of surfaces which have the highest susceptibility to dental caries (occlusal surfaces of first molars and buccal surfaces of lower first molars), the susceptibility Group 2 consisted of 12 surfaces (occlusal surfaces of second molars, buccal surfaces of lower second molars, occlusal surfaces of all second premolars and palatal surfaces of upper first molars), the susceptibility Group 3 consisted of 20 surfaces (occlusal surfaces of first premolars, palatal surfaces of upper lateral incisors, proximal surfaces of first molars, lingual surfaces of lower first molars, buccal surfaces of upper first molars and palatal surfaces of upper second molars), the susceptibility Group 4 included all other 90 surfaces. The dental caries experience was calculated separately for each surface-based susceptibility group and used as continuous variable in the analyses.

### Statistical analyses

Statistical analyses were performed employing the Statistical Package for the Social Sciences (SPSS, Version 26.0, IBM, Armonk, NY, USA). Participants with missing values in any of the study variables were excluded. Chi-square and Mann-Whitney U tests compared sociodemographic characteristics and oral health-related behaviors among participants stratified into a lower-level fluoride < 0.7 ppm and a higher-level fluoride ≥ 0.7 ppm in the drinking water (Table 1). Kruskal-Wallis test was used to compare median differences in dental caries experience and its components weighted by the number of surfaces in each caries surface-based susceptibility hierarchy group (Table 2).

**Table 1:** Distribution of potential determinants and dental caries experience among 35–74-year-olds (N = 946) and stratified by fluoride levels in the drinking water

Determinants		Fluoride level in the drinking water			Total
		Lower-level < 0.7 ppm		Higher-level ≥ 0.7 ppm	
*Socio-demographic characteristics*		748 (100 %)	198 (100 %)		946 (100%)
Sex	Female	490 (66)	138 (70)		628 (66)
	Male	258 (34)	60 (30)		318 (34)
Age*	Mean (SD) Median (IQR)	52.2 (11.8) 52 (21)	54.8 (11.0) 56 (20)		52.8 (11.7) 53 (40)
Education	Higher: 4 years or more	102 (14)	19 (10)		121 (13)
	Higher: less than 4 years	383 (51)	98 (49)		481 (51)
	Secondary school or less	263 (35)	81 (41)		344 (36)
Residency	Urban	553 (74)	149 (75)		702 (74)
	Peri-urban/rural	195 (26)	49 (25)		244 (26)
*Oral health-related behaviours*					
Tooth brushing frequency	Twice a day or more	368 (49)	102 (52)		470 (50)
	Once a day or less	380 (51)	96 (48)		476 (50)
Using fluoridated toothpaste	Yes	401 (54)	107 (54)		508 (54)
	No	113 (15)	30 (15)		143 (15)
	Don’t know	234 (31)	61 (31)		295 (31)
Consumption of sugar-containing foods**	Low	196 (26)	63 (32)		259 (28)
	Moderate	323 (43)	96 (48)		419 (44)
	High	229 (31)	39 (20)		268 (28)
Last dental visit	12 months ago or less	544 (73)	144 (73)		688 (73)
	More than 12 months ago	204 (27)	54 (27)		258 (27)
*Dental caries experience*					
D_3_ MFS	Mean (SD) Median (IQR)	59.0 (32.8) 55 (50)	55.0 (30.6) 51 (46)		58.1 (32.4) 54 (49)
D_3_ S	Mean (SD) Median (IQR)	2.6 (6.2) 1 (2)	2.1 (3.3) 1 (3)		2.5 (5.7) 1 (3)
MS	Mean (SD) Median (IQR)	27.8 (32.1) 15 (35)	24.1 (27.2) 15 (30)		27.0 (31.2) 15 (34)
FS	Mean (SD) Median (IQR)	28.5 (19.7) 26 (28)	28.8 (18.7) 27 (27)		28.6 (19.5) 26 (28)

*p < 0.05 according to Mann–Whitney U test

**p < 0.05 according to Chi-square test

**Table 2:** Proportion of D_3_S (decayed), MS (missing), FS (filled) and total D_3_MFS (decayed, missing, filled) surfaces in caries surface-based susceptibility hierarchy groups weighted by the number of surfaces in each group

Caries Index components	Caries surface-based susceptibility hierarchy										
	Group 1 6 surfaces			Group 2 12 surfaces		Group 3 20 surfaces		Group 4 90 surfaces		Total 128 surfaces	
D_3_ S	30%a		0.2 (0.6) 0 (0)	32%a	0.4 (1.0) 0 (0)	22%b	0.4 (1.3) 0 (0)	16%b	1.5 (3.9) 0 (1)	100%	2.5 (5.7) 1 (3)
MS	36%a		2.6 (2.2) 2 (4)	24%b	3.4 (3.7) 2 (6)	26%b	6.3 (5.8) 6 (9)	14%c	14.8 (21.2) 7 (18)	100%	27.0 (31.2) 15 (34)
FS	31%a		2.2 (1.8) 2 (3)	31%a	4.5 (2.9) 4 (5)	22%b	5.3 (4.0) 5 (6)	16%c	16.7 (14.2) 13 (20)	100%	28.6 (19.5) 26 (28)
D_3_ MFS	33%a		5.0 (1.4) 5 (2)	28%b	8.3 (3.0) 9 (5)	24%c	12.0 (5.6) 12 (8)	15%d	32.9 (24.3) 28 (35)	100%	58.1 (32.4) 54 (49)

Mean (SD) and median (interquartile range) are also presented in caries surface-based susceptibility hierarchy groups

The same letter indicates a non-significant association between the same components of the D_3_MFS index

Kruskal–Wallis test was used for the comparisons among caries surface-based susceptibility hierarchy groups

Univariable and multivariable linear regression analyses associated fluoride level in the drinking water and dental caries experience in four surface-based susceptibility hierarchy groups. The association in multivariable linear regression analysis was adjusted for all potential determinants. The testing of multicollinearity was based upon tolerance and VIF values [17]. The goodness of model fit was indicated by R^2^ [17]. For robust confidence intervals and significant tests of the model parameters, which do not rely on normality assumption, bootstrap option was used. The level of significance was set at *p* < 0.050 and β coefficients and 95% confidence intervals (CIs) are presented.

## Results

In our sample, 748 (79%) participants resided in areas with lower-level fluoride (< 0.7 ppm) and 198 (21%) in areas with higher-level fluoride (≥ 0.7 ppm) in the drinking water. A higher proportion of younger than of older participants resided in areas with lower-level fluoride in the drinking water (Table 1). In addition, a higher proportion of participants residing in areas with lower-level fluoride in the drinking water had moderate and high consumption of sugar-containing foods (Table 1). Otherwise, the study sample was similar when stratified by the level of fluoride in the drinking water.

The mean (standard deviation [SD]) D_3_MFS among 35–74-year-olds was 58.1 (32.4), median (interquartile range [IQR]) 54 (49) (Table 2). Of all surfaces with dental caries experience, the highest proportion was in Group 1 (33%) followed by Group 2 (28%), Group 3 (24%) and Group 4 (15%) when weighted by the number of surfaces in each surface-based susceptibility hierarchy group (Table 2).

According to multivariable linear regression analysis, higher-level fluoride in the drinking water (≥ 0.7 ppm vs < 0.7 ppm) statistically significantly associated with lower D_3_MFS scores in all surface-based susceptibility hierarchy groups; Group 1: β = − 0.23, *p* = 0.035 (95%CI − 0.44; − 0.001), Group 2: β − 0.44, *p* = 0.034 (95 %CI − 0.82; − 0.07), Group 3: β = − 1.14, *p* = 0.002 (95 %CI − 1.88; − 0.41) and Group 4: β = − 6.28, *p* = 0.001 (95 %CI − 9.29; − 3.30) (Table 3).

**Table 3:** Associations between D_3_MFS (decayed, missing, filled surfaces) in the caries surface-based susceptibility hierarchy groups and their potential determinants, according to univariable and multivariable linear regression models with robust confidence intervals (95%CI) and significance (*p* values) among 35–74-year-olds (N = 946)

Determinants		Caries experience (D_3_MFS)														
		Group 1			Group 2				Group 3				Group 4			
		Crude β *p *value (95%CI)	Adjusted β *p *value (95%CI)		Crude β *p *value (95% CI)	Adjusted β *p *value (95% CI)			Crude β *p *value (95%CI)		Adjusted β *p *value (95%CI)		Crude β *p *value (95% CI)		Adjusted β *p *value (95%CI)	
*Socio-demographic characteristics*																
Fluoride level in drinking water	Lower-level < 0.7 ppm	Ref	Ref		Ref	Ref			Ref		Ref		Ref		Ref	
	Higher-level ≥ 0.7 ppm	− 0.15, 0.194 (− 0.38; 0.09)	− 0.23, 0.035 (− 0.44; − 0.001)		− 0.13, 0.615 (− 0.61; 0.36)	− 0.44, 0.034 (− 0.82; − 0.07)			− 0.49, 0.267 (− 1.4; 0.38)		− 1.14, 0.002 (− 1.88; − 0.41)		− 3.21, 0.098 (− 7.02; 0.59)		− 6.28, 0.001 (− 9.29; − 3.30)	
Age/10 years	Continuous	0.29, 0.0001 (0.22; 0.36)	0.28, 0.001 (0.20; 0.36)		2.00, 0.0001 (1.05; 1.35)	1.20, 0.001 (1.05; 1.35)			2.47, 0.0001 (2.21; 2.74)		2.42, 0.001 (2.16; 2.66)		12.29, 0.0001 (11.21; 13.36)		12.05, 0.001 (10.84; 13.23)	
Sex	Males	Ref	Ref		Ref	Ref			Ref		Ref		Ref		Ref	
	Females	0.47, 0.0001 (0.28; 0.65)	0.50, 0.001 (0.30; 0.72)		1.17, 0.0001 (0.76; 1.59)	1.28, 0.001 (0.86; 1.73)			1.61, 0.0001 (0.86; 2.35)		1.80, 0.001 (1.11; 2.47)		3.94, 0.018 (0.67; 7.22)		4.98, 0.003 (2.36; 7.95)	
Education	Higher: 4 years or more	Ref	Ref		Ref	Ref			Ref		Ref		Ref		Ref	
	Higher: < 4 years	0.36, 0.009 (0.09; 0.64)	0.12, 0.408 (− 0.16; 0.40)		0.89, 0.005 (0.28; 1.51)	− 0.12, 0.691 (− 0.73; 0.49)			2.38, 0.0001 (1.30; 3.47)		0.36, 0.426 (− 0.62; 1.35)		7.91, 0.001 (3.18; 12.65)		− 2.09, 0.231 (− 5.58; 1.42)	
	Secondary school or less	0.50, 0.001 (0.22; 0.79)	0.23, 0.141 (− 0.11; 0.57)		1.46, 0.0001 (0.82; 2.10)	0.24, 0.403 (− 0.45; 0.93)			3.80, 0.0001 (2.67; 4.93)		1.23, 0.025 (0.06; 2.31)		16.17, 0.0001 (11.25; 21.09)		2.80, 0.158 (− 1.55; 6.93)	
Residency	Peri-urban/rural	Ref	Ref		Ref	Ref			Ref		Ref		Ref		Ref	
	Urban	− 0.33, 0.001 (− 0.52; − 0.13)	− 0.12. 0.283 (− 0.09; 0.30)		− 0.73, 0.002 (− 1.19; − 0.28)	− 0.08, 0.690 (− 0.50; 0.33)			− 1.67, 0.0001 (− 2.48; − 0.87)		− 0.37, 0.317 (− 1.08; 0.31)		− 6.75, 0.0001 (− 10.27; − 3.23)		− 1.35, 0.406 (− 4.43; 1.71)	
Oral health-related behaviors																
Tooth brushing frequency	Twice a day or more	Ref	Ref		Ref	Ref			Ref		Ref		Ref		Ref	
	Once a day or less	0.07, 0.446 (− 0.11; 0.24)	0.10, 0.283 (− 0.9; 0.30)		0.32, 0.116 (− 0.08; 0.72)	0.40, 0.028 (0.05; 0.75)			0.78, 0.030 (0.07; 1.49)		0.63, 0.060 (− 0.02; 1.30)		4.24, 0.007 (1.15; 7.33)		2.21, 0.094 (− 0.71; 4.96)	
Using fluoridated toothpaste	Yes	Ref	Ref		Ref	Ref			Ref		Ref		Ref		Ref	
	No	0.06, 0.668 (− 0.20; 0.31)	− 0.04, 0.708 (− 0.27; 0.18)		0.49, 0.095 (− 0.09; 1.07)	0.09, 0.704 (− 0.41; 0.59)			1.02, 0.053 (− 0.01; 2.05)		0.11, 0.808 (− 0.72; 0.94)		8.68, 0.0001 (4.21; 13.16)		4.23, 0.029 (0.52; 8.21)	
	Don’t know	0.10, 0.302 (− 0.09; 0.30)	0.03, 0.767 (− 0.15; 0.21)		0.17, 0.469 (− 0.28; 0.61)	− 0.12, 0.569 (− 0.52; 0.28)			0.51, 0.208 (− 0.29; 1.31)		− 0.09, 0.820 (− 0.71; 0.52)		4.46, 0.010 (1.10; 8.02))		1.73, 0.230 (− 0.98; 4.66)	
Consumption of sugar- containing foods	Low	Ref	Ref		Ref	Ref			Ref		Ref		Ref		Ref	
	Moderate	− 0.01, 0.994 (− 0.21; 0.21)	0.09, 0.406 (− 0.11; 0.29)		− 0.08, 0.784 (− 0.56; 0.41)	0.23, 0.307 (− 0.21; 0.60)			− 0.23, 0.595 (− 1.10; 0.63)		0.45, 0.226 (− 0.27; 1.12)		0.50, 0.794 (0− 3.27; 4.27))		4.06, 0.007 (0.72; 7.17)	
	High	0.07, 0.586 (− 0.17; 0.30)	0.23, 0.067 (0.00; 0.46)		− 0.04, 0.894 (− 0.57; 0.50)	0.55, 0.038 (0.02; 1.08)			− 0.17, 0.734 (− 1.12; 0.79)		0.96, 0.031 (0.07; 1.76)		− 0.49, 0.818 (− 4.65; 3.67)		4.67, 0.011 (1.33; 8.10)	
Last dental visit	12 months ago or less	Ref	Ref		Ref	Ref			Ref		Ref		Ref		Ref	
	More than 12 months ago	0.04, 0.682 (− 0.16; 0.24)	0.11, 0.302 (− 0.08; 0.31)		0.02, 0.925 (− 0.43; 0.47)	0.35, 0.119 (− 0.07; 0.80)			− 0.22, 0.591 (− 1.02; 0.58)		0.49, 0.174 (− 0.17; 1.09)		− 5.50, 0.002 (− 8.97; − 2.04)		− 1.87, 0.230 (− 4.97; 0.91)	
R^2^			0.102			0.247			0.310		0.310				0.387	

Ref, reference category

## Discussion

The current nationally representative cross-sectional adult study confirmed the hierarchy-based susceptibility hypothesis and demonstrated that the proportion of dental caries experience was the highest in the most susceptible surface-based group (Group 1) followed by the less susceptible groups (Groups 2 and 3), and the lowest proportion of dental caries experience was observed in the least susceptible surface-based group (Group 4) when weighted by the number of surfaces in each group. Moreover, the higher-level fluoride (≥ 0.7 ppm vs < 0.7 ppm) in the drinking water was significantly and consistently associated with the lower dental caries experience in all surface-based susceptibility hierarchy groups.

Following the WHO criteria, dental caries was recorded clinically only at the dentin level, consequently, the total dental caries experience could be underestimated. However, these diagnosis-related limitations should not impact the association between fluoride level in the drinking water and dental caries experience.

As a study outcome, we used dental caries experience measured as the D_3_MFS index. It has been debated if the D_3_MFS index including M component (vs D_3_FS), is the most appropriate caries measure for adults [18]. Fluoride in the drinking water impacts population at large as well as all caries experience-related components, therefore we considered it reasonable also to include the M component [19, 20]. Moreover, the DMF index is the most commonly used measure when assessing the effect of fluoride level in the drinking water on dental caries [21].

The present study employed a cross-sectional study design, therefore causality could not be inferred. When examining the association between fluoride levels in the drinking water and dental caries experience, we adjusted for several confounding factors. Also, when public health prevention measures, such as fluoride level in the drinking water, are to be evaluated, cross-sectional studies are considered appropriate [22]. We had no information for how long our participants resided in the area with the same fluoride level in drinking water, which is the major limitation of the present study. However, based on the survey among 16–64-year-old Lithuanians, only 13% reported having ever changed their living place [23]. In addition, except for the use of fluoridated toothpaste, no information about systemic and topically applied fluoride supplements was collected.

The surface-based susceptibility hierarchy was previously evaluated only in children [10]. The present study demonstrated that each less susceptible surface-based susceptibility group consistently presented lower proportion of D_3_MFS compared to more susceptible groups. This finding suggests that caries-based surface susceptibility hierarchy may also be relevant to adults and confirms our first hypothesis. The utility of the surface-based susceptibility hierarchy could be used for planning preventive strategies; if all surfaces in the same hierarchy group would behave in the same way, then the resistance of the whole group of surfaces would also be similar. For example, if one surface susceptibility group would associate with fluoride level in the drinking water and the other one with consumption of sugar-containing foods, then the preventive measures would be respective to that group [10].

Even though, specific genes, as caries determinants, were previously analyzed using the surface susceptibility group approach [24–27], the evidence for other caries determinants presented by the surface-based susceptibility groups is currently lacking.

In the present study, higher-level fluoride in the drinking water was associated with lower caries experience in all surface-based susceptibility hierarchy groups, consequently our second study hypothesis was not supported by present findings. This finding is in line with an earlier adolescent study demonstrating that fluoride may have an equal preventive effect on different surface types [18]. Therefore, based on our findings, the surface-based susceptibility hierarchy groups approach does not seem to be useful for planning preventive fluoride strategies for adults.

In general, there is scarce and inconsistent evidence about the association between fluoride levels in drinking water and dental caries in adults. The recommended threshold for water fluoridation is 0.5–1.0 ppm [28]. Recently it has been suggested to reduce the “optimum” water fluoridation level to 0.7 ppm that was supported by evidence concerning the preventive effect on dental caries among children [16].

In Lithuania, some regions have naturally fluoridated drinking water ranging between 0.15 and 1.46 ppm. Regarding the effect of the fluoridated water on caries in adults, the Cochrane systematic review did not identify adult studies fitting their inclusion criteria [29]. However, the authors were criticized for employing too strict inclusion criteria [20]. An earlier systematic review and meta-analysis concluded that fluoridated water had the potential to reduce dental caries among adults of all ages [30]. Earlier studies performed in Australia, Brazil, the UK, and the USA showed that fluoride in the drinking water had a preventive effect against caries in adults [31–34]. However, there is a lack of contemporary evidence of the fluoridated water’s effect on dental caries. The present cross-sectional study showed the association between different fluoride levels in the drinking water and dental caries experience among adults. To the best of our knowledge, our study is one of the few contemporary adult studies which demonstrated that study participants residing in areas with 0.7 ppm or higher levels of fluoride in the drinking water associated with lower dental caries experience. Our findings are in line with another national representative adult study performed in Australia showing the inverse association between water fluoridation and caries experience [35].

## Conclusions

This adult cross-sectional study demonstrated that dental caries experience followed the hierarchy of surface-based susceptibility. The higher-level fluoride in the drinking water associated with lower dental caries experience in adults and this was observed in all surface-based susceptibility groups. However, there is a need for more adult studies to validate the present findings, especially adopting a longitudinal study design, to examine the potential of surface-based susceptibility hierarchy.

## Data Availability

The dataset analyzed during the current study is available from the corresponding author upon reasonable request.

## Abbreviations

CI: Confidence interval
D_3_MFS: Total number of decayed, missed, and filled surfaces
IQR: Interquartile range
NLOHS: National Lithuanian Oral Health Survey
SD: Standard deviation
WHO: World Health Organization
